# A psychological dynamics model of AI-supported learning: examining the mediating and moderating effects on preservice early childhood teachers’ instructional design ability

**DOI:** 10.3389/fpsyg.2026.1776056

**Published:** 2026-05-22

**Authors:** Feifei Teng

**Affiliations:** School of Literature and Journalism, Zhengzhou Business University, Gongyi, Henan, China

**Keywords:** AI feedback, AI scaffolding, AI-supported teacher education, creative self-efficacy, instructional design ability, professional identity, self-regulated learning

## Abstract

**Introduction:**

Generative AI is reshaping higher education by acting not only as a learning tool but also as a psychological catalyst for self-regulation and creative expression. Drawing on Self-Regulated Learning Theory, Social Cognitive Theory, and Self-Determination Theory, this study examines how AI feedback quality and AI scaffolding influence the instructional design ability of preservice early childhood education students through the mediating roles of self-regulated learning (SRL) and creative self-efficacy (CSE), as well as the moderating role of professional identity in early childhood education (PIEC).

**Methods:**

A cross-sectional online survey was conducted with 439 undergraduate students majoring in early childhood education at universities in mainland China. All constructs were measured with validated 7-point Likert scales. The hypothesized model was tested using Partial Least Squares Structural Equation Modeling (PLS-SEM) in SmartPLS 4.0, with 5,000 bootstrap resamples to assess direct, indirect (mediation), and interaction (moderation) effects.

**Results:**

Both AI feedback quality and AI scaffolding significantly enhanced SRL and CSE, which in turn improved instructional design ability. SRL and CSE each exhibited significant mediating effects, with SRL demonstrating the stronger indirect influence. Professional identity displayed a dual moderating pattern: it strengthened the effects of AI feedback on SRL and CSE but weakened the effects of AI scaffolding.

**Discussion:**

These findings reposition AI as a psychological facilitator that triggers strategic and creative processes rather than merely a technical tool. The study contributes a dual-process model of AI-supported learning and a professional congruence moderation framework, offering implications for teacher preparation, AI tool selection, and instructional design training. Limitations and directions for future research are discussed.

## 1 Introduction

In recent years, the theoretical positioning of Artificial Intelligence (AI) in higher education has shifted from the early stage of Computer-Assisted Learning (CAL) to a “feedback–regulation–creation” learning paradigm centered on Generative AI (GenAI). This transformation not only redefines how learners interact with knowledge but also reshapes their roles within the self-regulated learning process—from passive recipients to co-regulators who co-construct their learning experiences with AI.

Through real-time feedback and adaptive support, AI can trigger learners’ reflection, strategic adjustment, and motivational maintenance, forming a dynamic, generative learning cycle (Lee and Moore, 2024; Qi et al., 2025). Furthermore, contextual scaffolding based on large language models has transcended the limits of traditional rule-based instruction, providing semantically rich, task-oriented guidance that enables learners to develop transferable professional knowledge in authentic instructional design contexts (Yin and Yin, 2024). Thus, AI is no longer merely a technological aid for learning but functions as a psychological catalyst that mediates the interaction among learning regulation, motivational maintenance, and creative performance.

However, the benefits of AI-supported learning do not occur automatically; they depend on the psychological transformation processes within learners. From an educational psychology perspective, the mechanisms underlying AI’s effects can be explained through three key theories: Self-Regulated Learning Theory (SRL), Social Cognitive Theory (SCT), and Self-Determination Theory (SDT).

First, SRL theory posits that learners actively manage their learning through a cyclical process of goal setting, strategic implementation, and self-reflection (Zimmerman, 2000b). When AI provides structured, constructive, and timely feedback, it can enhance learners’ strategic adjustments and goal orientation within this cycle (Lan and Zhou, 2025). Second, SCT emphasizes that self-efficacy is a central predictor of behavioral motivation (Bandura, 1997), and Creative Self-Efficacy (CSE) determines whether learners have the confidence to engage in innovation and problem solving (Tierney and Farmer, 2002; Unal and Taşar, 2021). By offering model examples and positive reinforcement, AI can strengthen learners’ creative confidence and persistence in action. Third, SDT proposes that autonomy, competence, and relatedness are the three basic psychological needs that underpin intrinsic motivation (Deci and Ryan, 2000). When AI-supported learning environments fulfill these needs, external stimuli can be internalized into sustained motivational energy and creative engagement (Ma and Chen, 2025).

Integrating these perspectives, the present study proposes an AI-Supported Psychological Energy Model, which conceptualizes how AI feedback facilitates learners’ professional growth through the psychological pathway of self-regulation → efficacy belief → creative instructional performance.

Nevertheless, this psychological transformation does not occur uniformly across all learners. Research has shown that professional identity plays a crucial moderating role in determining the internalization of AI-supported learning (Beijaard et al., 2004; Izadinia, 2013). For early childhood education (ECE) students, Professional Identity in Early Childhood Education (PIEC) represents not only the core of professional socialization but also a psychological bridge that transforms external technological support into intrinsic professional growth (Cai et al., 2022; Mayangsari et al., 2024). When learners possess a strong sense of professional identity and value alignment with their future teaching roles, they are more likely to satisfy their needs for autonomy and competence, thereby enhancing the positive effects of AI support on self-regulation and creative self-efficacy. Empirical findings further indicate that preservice teachers’ professional identity is significantly correlated with their learning engagement, adaptability, and innovative behavior (Zhang et al., 2016), suggesting that professional identity amplifies the efficiency with which learning resources are transformed into professional competence.

Accordingly, this study conceptualizes a core pathway of AI support → self-regulation → creative self-efficacy → instructional design ability, incorporating professional identity as a moderating construct to reveal the psychological variations and transformation mechanisms underlying AI-supported learning outcomes. This model not only extends the theoretical discussion of AI applications to the psychological domain but also provides an initial framework for an AI-integrated Motivation Theory in teacher education.

## 2 Literature review and research hypotheses

### 2.1 Theoretical foundations

This study draws primarily on Self-Regulated Learning Theory (SRL) and Social Cognitive Theory (SCT) to explain how AI feedback and AI scaffolding promote the development of instructional design ability among preservice early childhood education students. These two frameworks, respectively, address (1) the regulation of learning processes and (2) the formation of efficacy beliefs.

#### 2.1.1 Self-regulated learning theory (SRL)

Self-Regulated Learning Theory emphasizes that learning is an active, cyclical, and controllable process (Zimmerman, 2000a). Learners are expected to engage in three iterative phases—forethought, performance control, and self-reflection—in which they set goals, monitor progress, and adjust strategies to achieve desired learning outcomes. According to Pintrich (2004), SRL integrates cognitive, motivational, and behavioral dimensions, enabling learners to flexibly adapt strategies according to task demands.

In AI-supported learning environments, real-time feedback and structured prompts provided by AI function as external regulatory resources, facilitating goal setting, strategy adjustment, and reflective thinking. When AI feedback is interpretable, constructive, and timely, it can significantly enhance learners’ self-monitoring capabilities and overall learning performance (Afzaal et al., 2024; Lan and Zhou, 2025).

Therefore, SRL theory elucidates the dynamic regulatory mechanism of the pathway “AI support → self-monitoring and strategic reflection → instructional design ability.” It explains how AI promotes the internalization of self-directed learning processes, thereby strengthening learners’ professional knowledge construction and design competence.

#### 2.1.2 Social cognitive theory (SCT)

Social Cognitive Theory (SCT) emphasizes the principle of reciprocal determinism, which posits that behavior, environment, and personal factors interact dynamically to shape learning and performance (Bandura, 1997). Among these factors, self-efficacy serves as the central construct influencing motivation and behavioral outcomes. It reflects an individual’s confidence in successfully performing a specific task and directly affects both the choice of action and the level of persistence.

Building on this concept, Tierney and Farmer (2002) introduced Creative Self-Efficacy (CSE), defined as learners’ belief in their ability to generate novel and valuable ideas. Subsequent research has confirmed that CSE is a significant predictor of creative performance (Nemeržitski and Heinla, 2020). In the context of AI-supported learning, examples, real-time feedback, and positive reinforcement provided by AI can enhance learners’ efficacy beliefs through vicarious learning and self-reinforcement mechanisms (Bandura, 1997). When learners develop confidence in effectively utilizing AI tools and transforming knowledge into creative outputs, they are more likely to demonstrate higher-order thinking and innovative instructional design competence.

Accordingly, SCT provides a psychological explanation for the pathway “AI support → Creative Self-Efficacy → Instructional Design Ability,” illustrating how AI promotes professional creative performance through the formation and strengthening of efficacy beliefs.

### 2.2 Relationships among AI feedback quality, self-regulated learning, and creative self-efficacy

The role of Artificial Intelligence (AI) in education has evolved from the automation of knowledge delivery to the dynamic regulation of learning processes. Within this paradigm, AI Feedback Quality (AIFQ) has emerged as a critical factor influencing both learners’ regulatory processes and efficacy beliefs. High-quality AI feedback is not only characterized by accuracy and timeliness but also by its constructiveness and actionability, which help learners identify errors, adjust strategies, and sustain intrinsic motivation (Shute, 2008; Lipnevich and Smith, 2009).

From a theoretical perspective, AIFQ can be regarded as an external regulatory resource that provides learners with informative and stimulating cues, thereby activating self-monitoring and strategic adjustment mechanisms. Through this process, AI feedback contributes to the enhancement of Self-Regulated Learning (SRL) and the development of Creative Self-Efficacy (CSE). In other words, constructive and actionable feedback serves not only as an instructional aid but also as a psychological scaffold that facilitates the internalization of self-regulatory and creative learning capacities.

#### 2.2.1 The relationship between AI feedback quality and self-regulated learning

According to Self-Regulated Learning Theory (Zimmerman, 2000a), learners actively manage their learning processes through a cyclical sequence of goal setting, strategic implementation, and self-reflection. Within this cycle, high-quality AI feedback can serve as a regulatory trigger, helping learners promptly detect errors, adjust strategies, and refine learning goals. Afzaal et al. (2024) demonstrated that informative and interpretable AI feedback enhances learners’ strategic awareness and precision in corrective actions, thereby strengthening their metacognitive monitoring abilities. Similarly, Lan and Zhou (2025) found that structured and real-time intelligent feedback fosters learners’ active engagement and deeper cognitive processing across the forethought, performance, and reflection phases of SRL.

Theoretically, the higher the quality of AI feedback, the more effectively learners can sustain positive motivation and strategy-oriented behaviors throughout the self-regulatory cycle. Pintrich (2004) posited that specific and meaningful feedback can be internalized as a cognitive resource, enhancing learners’ sense of control and mastery. In contrast, vague or delayed feedback may weaken self-monitoring and reduce sustained engagement. Empirical studies further support this view—immediate and constructive AI feedback has been shown to significantly improve learners’ planning ability and reflective depth (Banihashem et al., 2025).

In the context of instructional design, preservice early childhood education students must integrate multiple layers of pedagogical factors, such as curriculum objectives, child characteristics, and instructional strategies. High-quality AI feedback provides concrete guidance and conceptual focus during this complex process, enabling learners to refine strategies, build effective self-regulatory models, and enhance professional knowledge construction and creative performance. Therefore, this study proposes the following hypothesis:

*H1*: AI feedback quality has a positive effect on preservice early childhood education students’ self-regulated learning.

#### 2.2.2 The relationship between AI feedback quality and creative self-efficacy

Creative Self-Efficacy (CSE), derived from Bandura’s (1997) Social Cognitive Theory, refers to individuals’ belief in their ability to generate novel and valuable ideas. This belief determines whether learners possess the confidence to experiment with innovative approaches and persist in improving their work, thereby predicting creative performance (Tierney and Farmer, 2002; Nemeržitski and Heinla, 2020). The formation of self-efficacy is grounded in three primary sources—mastery experience, vicarious experience, and social persuasion (Bandura, 1997). The socially interactive nature of AI feedback allows it to simultaneously satisfy these three sources, making it a critical psychological catalyst for the development of CSE.

First, high-quality AI feedback fosters mastery experience by providing accurate guidance and timely responses. When learners experience success through iterative revisions and improvements, their belief in their own creative ability is strengthened (Nemeržitski and Heinla, 2020). Second, AI-generated examples and generative suggestions provide vicarious experiences, enabling learners to observe and model effective creative strategies—a process consistent with Bandura’s concept of observational learning. Third, the positive reinforcement and semantic encouragement embedded in AI feedback and collaboration offer a form of social persuasion. As learners shift from passively receiving feedback to co-creating with AI, their sense of agency and creative confidence increases (Jeong and Jeong, 2025; McGuire et al., 2024).

In summary, high-quality AI feedback not only provides concrete instructional guidance but also cultivates creative belief through mechanisms of social interaction and contextual reinforcement. By integrating mastery experience, vicarious experience, and social persuasion, AI feedback strengthens learners’ confidence in their creative potential and their willingness to act upon it. Therefore, this study proposes the following hypothesis:

*H2*: AI feedback quality has a positive effect on preservice early childhood education students’ creative self-efficacy.

### 2.3 The relationship between AI scaffolding use, self-regulated learning, and creative self-efficacy

#### 2.3.1 The relationship between AI scaffolding use and self-regulated learning

The Scaffolding Theory, proposed by Wood et al. (1976), posits that external support enables learners to accomplish tasks within their Zone of Proximal Development (ZPD) that would otherwise exceed their independent capabilities. Within the context of AI-supported learning, this concept has evolved into intelligent scaffolding, wherein AI provides structured prompts, modeled examples, and adaptive guidance to help learners progressively construct knowledge and skills (Hmelo-Silver et al., 2007; Pea, 2018).

From the perspective of Self-Regulated Learning Theory (SRL) (Zimmerman, 2000a), AI scaffolding plays a vital role across all three SRL phases. During the forethought phase, AI offers examples and suggested action paths that guide learners in setting clear goals and plans. In the performance phase, AI facilitates real-time monitoring and strategy adjustment through task decomposition and process cues. Finally, in the self-reflection phase, AI provides evaluative feedback and improvement suggestions that strengthen metacognitive reflection and the internalization of self-regulatory strategies (Afzaal et al., 2024; Banihashem et al., 2025). Through this dynamic process, learners gradually shift from external guidance to internal control, forming a stable and autonomous pattern of self-regulated learning.

Furthermore, AI scaffolding can satisfy learners’ three basic psychological needs—competence, autonomy, and relatedness—as identified in Self-Determination Theory (Deci and Ryan, 2000). Empirical studies have shown that integrating AI with scaffolding significantly enhances learners’ need satisfaction and overall learning performance (Ma and Chen, 2025). Similar effects have also been observed in physical education and educational technology contexts (Hsia et al., 2025; Wang et al., 2025).

In summary, AI scaffolding not only provides task-oriented technical support but also promotes learners’ strategic awareness, metacognitive monitoring, and intrinsic motivation, thereby reinforcing the process of self-regulated learning. Therefore, this study proposes the following hypothesis:

*H3*: AI scaffolding use has a positive effect on preservice early childhood education students’ self-regulated learning.

#### 2.3.2 The relationship between AI scaffolding use and creative self-efficacy

According to Social Cognitive Theory (Bandura, 1997), self-efficacy beliefs are central determinants of behavioral motivation and persistence. Within this framework, Creative Self-Efficacy (CSE) refers to learners’ confidence in their ability to generate novel and valuable ideas (Tierney and Farmer, 2002). AI scaffolding, through structured guidance, modeled examples, and real-time feedback, supports the formation of this belief on both the task-oriented and psychological levels.

Theoretically, AI scaffolding aligns with the three primary sources of efficacy beliefs proposed by Bandura (1997): mastery experience, vicarious experience, and social persuasion.

First, the step-by-step guidance and adaptive feedback provided by AI allow learners to accumulate successful experiences through iterative revisions, thereby reinforcing their sense of mastery and confidence in their creative capability (Hmelo-Silver et al., 2007). Second, AI-generated examples and generative suggestions serve as vicarious experiences, helping learners observe effective design strategies and reduce uncertainty in creative tasks (Pea, 2018). Finally, the positive reinforcement and semantic encouragement offered by AI systems act as a form of social persuasion, providing emotional support and recognition that enhance learners’ willingness to engage in creative experimentation (Jeong and Jeong, 2025; McGuire et al., 2024).

In summary, AI scaffolding not only provides external support for knowledge construction but also strengthens creative beliefs through these three psychological mechanisms. As AI-guided learners transition from task execution to autonomous creation, their internalized sense of “I can create” becomes a sustained driver of innovation and professional growth. Therefore, this study proposes the following hypothesis:

*H4*: AI scaffolding use has a positive effect on preservice early childhood education students’ creative self-efficacy.

### 2.4 The effects of self-regulated learning and creative self-efficacy on instructional design ability

#### 2.4.1 The relationship between self-regulated learning and instructional design ability

Self-Regulated Learning (SRL) is a key psychological mechanism underlying professional competence development, particularly in instructional design tasks that integrate theory, practice, and creativity. According to Zimmerman (2000a) and Pintrich (2004), SRL is an active, cyclical process comprising three phases: forethought (goal setting and planning), performance control (strategy use and monitoring), and self-reflection (evaluation and revision). Learners with high levels of self-regulation can maintain motivation, flexibly adjust strategies, and achieve deeper conceptual understanding throughout the learning process.

In the context of early childhood education instructional design, students must integrate curriculum objectives, child characteristics, and pedagogical strategies—tasks that demand continuous metacognitive monitoring and strategic reflection. When learners effectively apply self-regulation strategies, they can optimize the plan–execute–reflect cycle, transforming theoretical knowledge into creative and practical teaching designs (Zimmerman, 2000a; Pintrich, 2004).

Recent studies have further indicated that SRL not only enhances academic performance but also predicts the innovation and professional quality of instructional design outcomes. In AI-supported environments, learners with strong self-regulation strategies utilize feedback more effectively and demonstrate higher levels of strategic awareness and creative productivity (Banihashem et al., 2025).

In summary, SRL enables preservice early childhood education students to proactively plan, monitor, and revise their instructional design processes, integrating diverse knowledge and experiences to strengthen both the creativity and professional sophistication of their teaching designs.

Therefore, this study proposes the following hypothesis:

*H5*: Self-regulated learning has a positive effect on preservice early childhood education students’ instructional design ability.

#### 2.4.2 The relationship between creative self-efficacy and instructional design ability

Creative Self-Efficacy (CSE), a core construct within Social Cognitive Theory (SCT), refers to an individual’s belief in their ability to generate novel and valuable ideas (Tierney and Farmer, 2002). According to Bandura (1997), self-efficacy influences the strength of motivation, persistence of effort, and psychological resilience when facing challenges. Learners with high CSE are more likely to engage in exploratory and innovation-oriented behaviors, maintaining a strong problem-solving drive even in complex tasks.

In the context of instructional design, CSE functions as a psychological engine that drives creative idea generation, conceptual integration, and strategic innovation. For preservice early childhood education students, this involves transforming theoretical knowledge into teaching designs that are developmentally appropriate and contextually relevant. Learners with higher CSE tend to exhibit openness, risk-taking, and proactive experimentation, adjusting their instructional frameworks to meet diverse educational demands (Nemeržitski and Heinla, 2020).

Empirical evidence supports the positive association between CSE and instructional design competence. Jeong and Jeong (2025) found that learners with high CSE demonstrate stronger generative thinking and cross-domain integration abilities in AI-supported environments. Similarly, McGuire et al. (2024) revealed that CSE enhances collaborative innovation outcomes by reinforcing learners’ creative confidence and self-belief. In other words, when learners firmly believe in their creative potential, they are more capable of transforming abstract ideas into concrete, actionable teaching designs.

In sum, Creative Self-Efficacy enables preservice early childhood education students to exhibit higher levels of creative thinking and practical design capability, fostering instructional innovation and pedagogical value through a continuous process of confidence building and exploratory engagement. Therefore, this study proposes the following hypothesis:

*H6*: Creative self-efficacy has a positive effect on preservice early childhood education students’ instructional design ability.

### 2.5 The mediating role of self-regulated learning

Self-Regulated Learning (SRL) serves as a key psychological mediator in AI-supported learning environments. According to Zimmerman (2000a) and Pintrich (2004), SRL is an active and cyclical process in which learners continuously set goals, monitor progress, and modify strategies across the three phases of forethought, performance, and reflection. Through this process, external learning resources are gradually internalized into self-directed behavioral patterns.

In AI-supported contexts, the real-time guidance and structured prompts provided by AI feedback and scaffolding can effectively promote learners’ strategic awareness and reflective thinking (Afzaal et al., 2024; Banihashem et al., 2025). Particularly, when AI feedback is clear, constructive, and actionable, it helps learners identify learning gaps and adjust strategies accordingly (Shute, 2008; Lipnevich and Smith, 2009). However, such positive effects are often not direct, but rather realized through learners’ cognitive interpretation, strategy use, and self-regulatory processes.

Theoretically, AI feedback quality enhances instructional design ability by fostering learners’ self-monitoring, strategy adjustment, and goal-directed behavior. In other words, SRL acts as the mechanism through which external AI support is transformed into internal learning performance (Lan and Zhou, 2025). Therefore, this study proposes the following hypothesis:

*H7*: Self-regulated learning mediates the relationship between AI feedback quality and instructional design ability.

According to Scaffolding Theory (Wood et al., 1976), external support enables learners to accomplish tasks within their Zone of Proximal Development (ZPD) that would otherwise be beyond their independent capabilities. In AI-supported learning contexts, AI scaffolding provides structured prompts, modeled examples, and step-by-step guidance, offering dynamic task support that helps learners progressively construct knowledge and strategies (Hmelo-Silver et al., 2007; Pea, 2018).

However, the educational benefits of scaffolding are not automatically realized; rather, they depend on the learner’s ability to internalize external guidance into self-regulatory processes. When learners actively engage with AI prompts—by setting goals, monitoring strategies, and reflecting on their progress—the external support is transformed into an internal driving force for continuous learning. Empirical findings have also shown that AI scaffolding indirectly enhances instructional design ability by promoting self-monitoring and strategic adjustment (Lan and Zhou, 2025).

Therefore, this study posits that Self-Regulated Learning (SRL) serves as the psychological mechanism through which AI scaffolding is converted into instructional design competence. Through the activation of self-regulatory processes, external scaffolding becomes internalized as sustained professional performance. Accordingly, the following hypothesis is proposed:

*H8*: Self-regulated learning mediates the relationship between AI scaffolding use and instructional design ability.

### 2.6 The mediating role of creative self-efficacy

Creative Self-Efficacy (CSE) serves as a crucial psychological bridge linking AI-based learning support with the development of professional competence. According to Social Cognitive Theory (Bandura, 1997), self-efficacy is a central determinant of behavioral motivation and persistence—it influences whether individuals are willing to engage in challenging tasks, think creatively, and continuously refine their strategies. Tierney and Farmer (2002) further identified CSE as a key psychological antecedent of innovative behavior and creative outcomes, determining the depth of exploration and flexibility of strategies that individuals employ when confronting open-ended tasks.

In AI-supported learning environments, the quality of AI feedback (AI Feedback Quality, AIFQ) can enhance learners’ creative beliefs through three core mechanisms of efficacy development—mastery experience, vicarious experience, and social persuasion (Bandura, 1997). High-quality AI feedback, characterized by clarity, constructiveness, and positive reinforcement, enables students to accumulate successful experiences during revision and creation, observe effective exemplars, and receive emotional support through real-time semantic encouragement (Shute, 2008). Collectively, these experiences strengthen learners’ confidence in their creative potential and their willingness to take initiative in innovative tasks.

Empirical evidence also indicates that AI-based support can indirectly enhance creative performance and design outcomes by improving learners’ creative self-efficacy (McGuire et al., 2024). In other words, CSE functions as a critical psychological mechanism through which external AI feedback is transformed into internal creative energy and instructional design competence. Accordingly, this study proposes the following hypothesis:

*H9*: Creative self-efficacy mediates the relationship between AI feedback quality and instructional design ability.

Secondly, regarding the relationship between AI scaffolding use and instructional design ability, the structured guidance and modeled examples provided by AI scaffolding enable learners to experience a gradual sense of competence growth and strategic mastery as they progress through complex tasks (Hmelo-Silver et al., 2007; Wood et al., 1976). When students successfully complete challenging tasks with the layered support of AI, they develop the belief that “I can create effective solutions.” This belief strengthens their creative self-efficacy, encouraging them to engage in greater innovation and demonstrate cognitive flexibility in subsequent instructional design processes.

Empirical evidence further supports this mechanism: AI scaffolding enhances creative outcomes and design performance by boosting learners’ self-efficacy and confidence in their creative potential (Jeong and Jeong, 2025). Accordingly, this study proposes the following hypothesis:

*H10*: Creative self-efficacy mediates the relationship between AI scaffolding use and instructional design ability.

### 2.7 The moderating role of professional identity in early childhood education

Professional Identity in Early Childhood Education (PIEC) represents a central psychological construct in the professional socialization of early childhood education students, reflecting their value beliefs, professional responsibility, and sense of belonging toward the role of an early childhood educator (Beijaard et al., 2004). According to Self-Determination Theory (SDT; Deci and Ryan, 2000), professional identity strengthens learners’ intrinsic motivation, enabling external supports—such as AI feedback and scaffolding—to be internalized into autonomous self-regulatory processes. Learners with a high level of professional identity are more likely to perceive external inputs as resources for professional growth rather than as external demands, thus demonstrating greater self-direction and sustained learning motivation. Conversely, those with a low sense of identity may lack the motivation to internalize external support into meaningful learning actions (Izadinia, 2013).

Within AI-supported learning contexts, professional identity functions as a key moderating variable that influences the extent to which learning processes are internalized. Specifically, in the relationship between AI feedback quality and self-regulated learning, students with strong professional identity are more inclined to actively interpret, assimilate, and apply AI feedback, transforming it into a valuable professional resource that enhances instructional design ability (Afzaal et al., 2024). In contrast, those with weaker professional identity may struggle to construct meaning from feedback or translate it into strategic learning actions, thereby limiting their self-regulatory development. Accordingly, this study proposes the following hypothesis:

*H11*: The positive effect of AI feedback quality on self-regulated learning is stronger when professional identity in early childhood education is higher.

Secondly, in the relationship between AI scaffolding use and self-regulated learning, students with high professional identity are more likely to perceive AI scaffolding as a facilitator of professional growth. Such learners actively engage with AI-provided guidance, examples, and strategic prompts, demonstrating greater initiative and responsibility during task execution, which in turn strengthens their self-regulatory learning processes. Conversely, those with low professional identity may rely on AI support in a passive or surface-level manner, showing limited engagement and reflection.

*H12*: The positive effect of AI scaffolding use on self-regulated learning is stronger when professional identity in early childhood education is higher.

Furthermore, in the relationship between AI feedback quality and creative self-efficacy, a strong sense of professional identity can amplify the perceived validation and psychological support that learners derive from AI feedback. When students possess a deep sense of belonging and value commitment to the early childhood education profession, they are more likely to interpret AI’s positive reinforcement and model-based suggestions as affirmations of their professional competence, thereby enhancing their creative confidence and willingness to innovate (Jeong and Jeong, 2025). In contrast, those with weaker professional identity may respond more indifferently to AI feedback, failing to internalize it as a source of confidence or creative motivation.

*H13*: The positive effect of AI feedback quality on creative self-efficacy is stronger when professional identity in early childhood education is higher.

Finally, in the relationship between AI scaffolding use and creative self-efficacy, professional identity also exerts an amplifying effect. Learners with high professional identity in early childhood education are more inclined to transform external guidance from AI into intrinsic motivation for creative practice. Within AI-supported learning environments, they develop the belief that “I can design innovative solutions that align with professional values and meet children’s developmental needs”, thereby strengthening their creative self-efficacy (Beauchamp and Thomas, 2009; Ye et al., 2025). Conversely, those with low professional identity may rely on AI assistance only superficially, lacking genuine engagement with creativity or professional value pursuit.

*H14*: The positive effect of AI scaffolding use on creative self-efficacy is stronger when professional identity in early childhood education is higher.

**Figure 1:**
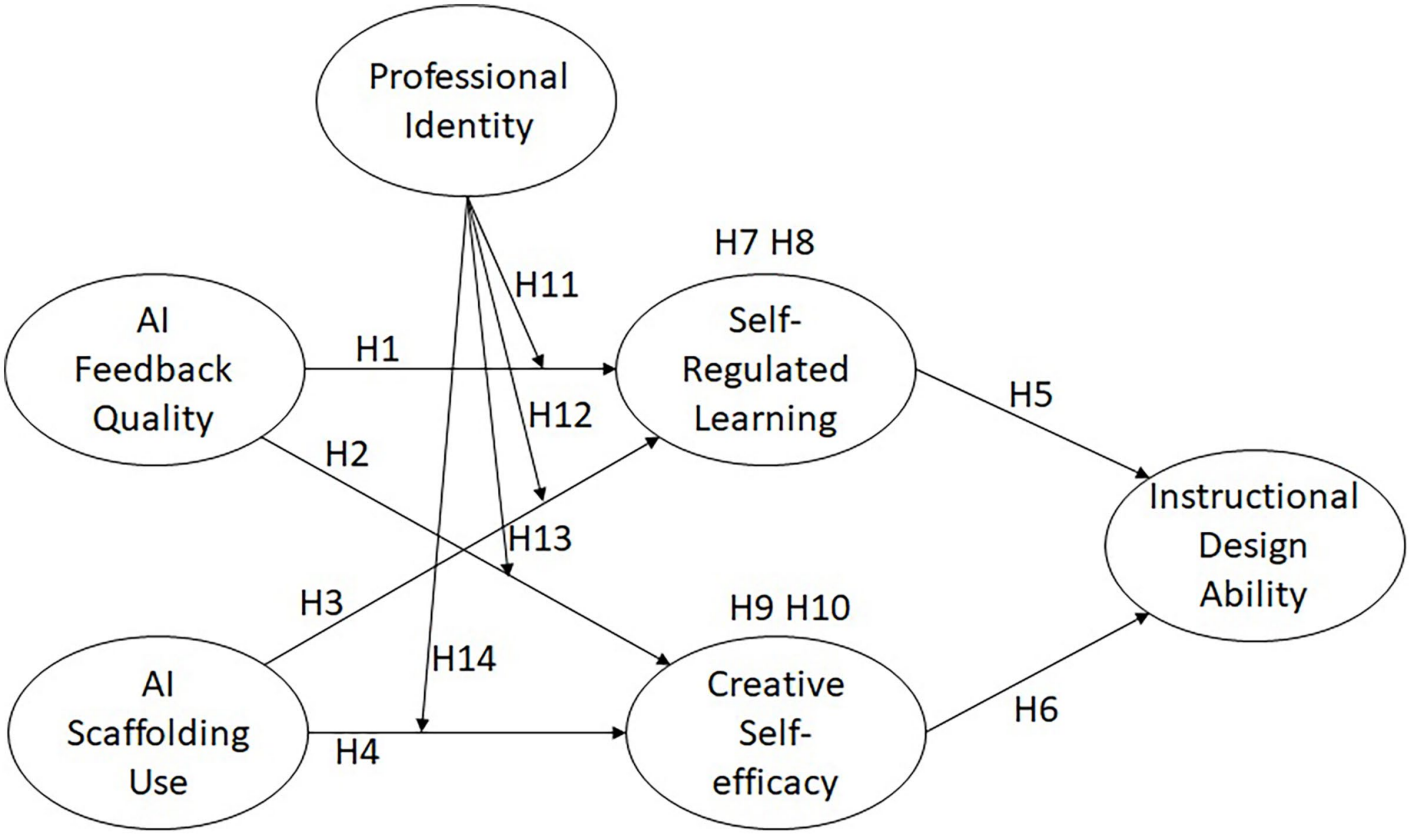
Research framework.

Based on the above hypotheses, this study proposes the research framework illustrated in Figure 1, which integrates the mediating roles of self-regulated learning and creative self-efficacy, as well as the moderating role of professional identity within the AI-supported psychological energy model.

## 3 Research methodology

### 3.1 Research participants and data collection

The participants of this study were undergraduate students majoring in Early Childhood Education (ECE) at universities in mainland China. Data were collected through an online questionnaire survey. The survey was distributed to students across multiple higher education institutions via university academic systems, student communities, and communication groups. According to the guideline proposed by Hair et al. (2019), the minimum sample size for Partial Least Squares Structural Equation Modeling (PLS-SEM) should satisfy the “10-times rule,” meaning that the sample should be at least 10 times the largest number of structural paths directed toward a particular construct. In this study’s research model, the maximum number of paths directed toward the mediating variables (i.e., from AI feedback quality and AI scaffolding use to self-regulated learning and creative self-efficacy) is four. Therefore, a minimum of 200 valid responses is required to meet this criterion. Additionally, based on the sample size table proposed by Krejcie and Morgan (1970), when the population consists of a large group of university students, the recommended minimum sample size is approximately 384. To ensure adequate statistical power and the robustness of the results, the final target sample size for this study was set between 400 and 500 participants.

### 3.2 Questionnaire design

This study employed a questionnaire survey as the primary data collection instrument. The questionnaire consisted of two main sections. The first section gathered demographic information, including gender, year level, experience in early childhood education internships, and experience in courses related to artificial intelligence (AI) or educational technology. The second section addressed the core research constructs, which were adapted from validated measurement scales in prior studies and contextualized to the application of AI in instructional design. The questionnaire included six key variables, each measured using a 7-point Likert scale (1 = strongly disagree, 7 = strongly agree).

(1) AI Feedback Quality (AIFQ) — measured students’ perceptions of the accuracy, clarity, timeliness, and usefulness of AI system feedback (Shute, 2008; Lipnevich and Smith, 2009).

(2) AI Scaffolding Use (AISU) — assessed the extent to which students received prompts, examples, and structured guidance from AI to construct knowledge and develop skills during instructional design tasks (Wood et al., 1976; Hmelo-Silver et al., 2007; Pea, 2018).

(3) Self-Regulated Learning (SRL) — evaluated students’ abilities to set goals, monitor progress, adjust learning strategies, and engage in self-reflection throughout the learning process (Zimmerman, 2000a; Pintrich, 2004).

(4) Creative Self-Efficacy (CSE) — measured students’ beliefs in their ability to generate novel and valuable ideas and apply them to instructional design tasks (Bandura, 1997; Tierney and Farmer, 2002).

(5) Instructional Design Ability (IDA) — assessed students’ capabilities to integrate educational objectives, child characteristics, and instructional strategies in designing and implementing innovative and effective teaching plans (Maribe, 2009; Koehler and Mishra, 2009; Reigeluth, 2013).

(6) Professional Identity in Early Childhood Education (PIEC) — evaluated students’ value identification, sense of responsibility, and professional belonging toward the role of early childhood educators (Beijaard et al., 2004; Beauchamp and Thomas, 2009; Izadinia, 2013).

### 3.3 Data analysis

Data analysis in this study was conducted using SmartPLS 4.0, applying the Partial Least Squares Structural Equation Modeling (PLS-SEM) approach to test the proposed theoretical model and hypotheses. First, SPSS 28.0 was used for data preprocessing and descriptive statistical analysis to examine data completeness, normality, and potential outliers. Subsequently, the measurement model was assessed for reliability and validity. Specifically, indicator loadings, Cronbach’s *α*, Composite Reliability (CR), and Average Variance Extracted (AVE) were evaluated to ensure internal consistency and convergent validity. Discriminant validity was further verified using the Fornell–Larcker criterion and Heterotrait–Monotrait ratio (HTMT). Next, the structural model was analyzed to test the hypothesized relationships among constructs. A bootstrapping procedure with 5,000 resamples was employed to determine the statistical significance of path coefficients. The model’s predictive and explanatory power was evaluated using Coefficient of Determination (*R*^2^), Effect Size (*f*^2^), and Predictive Relevance (*Q*^2^) indices. Furthermore, the mediating and moderating effects were tested following the procedures recommended by Preacher and Hayes (2008). The indirect effects were considered significant when the 95% bias-corrected confidence interval (CI) did not include zero. All analytical procedures were conducted in accordance with the guidelines proposed by Hair et al. (2019) to ensure the robustness and theoretical soundness of model estimation.

## 4 Research results

### 4.1 Analysis of demographic data

A total of 439 valid responses were collected. Females accounted for 74.7% (*n* = 328) and males 25.3% (*n* = 111). Most respondents were seniors (39.2%), followed by sophomores (23.5%), freshmen (20.5%), and juniors (16.9%). Regarding practicum experience, 58.1% (*n* = 255) had completed an early childhood education internship, while 41.9% (*n* = 184) had not. In addition, 39.0% (*n* = 171) had taken AI or educational technology courses, whereas 61.0% (*n* = 268) had not. Overall, the sample was predominantly female and upper-year students, with most having internship experience but relatively limited exposure to AI-related coursework, as shown in Table 1.

**Table 1:** Presents the demographic characteristics of the participants.

Category	Group	Frequency distribution	Percentage
Gender	Male	111	25.3
	Female	328	74.7
What is your current year of study?	Freshman	90	20.5
	Sophomore	103	23.5
	Junior	74	16.9
	Senior	172	39.2
Do you have any internship experience in early childhood education?	Yes	255	58.1
	No	184	41.9
Have you taken any courses related to artificial intelligence or educational technology?	Yes	171	39.0
	No	268	61.0

A total of 439 valid responses were collected. Females accounted for 74.7% (*n* = 328), while males accounted for 25.3% (*n* = 111). The higher proportion of female participants reflects the actual gender distribution in early childhood education (ECE), which is widely recognized as a female-dominated field in both national and international contexts (OECD, 2020; UNESCO, 2019). Therefore, the gender composition of the sample is consistent with the structural characteristics of the target population rather than a result of sampling bias. In addition, this study adopted a purposive sampling strategy focusing on preservice early childhood education students. The primary objective was to examine the structural relationships among key psychological constructs within a specific professional education context, rather than to estimate population parameters. As noted by Hair et al. (2019), Partial Least Squares Structural Equation Modeling (PLS-SEM) is particularly suitable for theory testing and does not require strict probabilistic sampling for generalization. Nevertheless, it should be noted that the findings of this study are primarily applicable to preservice teachers in early childhood education programs. Future research is encouraged to include more gender-balanced samples or extend the investigation to other teacher education disciplines to further enhance generalizability.

### 4.2 Measurement model assessment

All constructs showed strong measurement quality. Item loadings ranged from 0.816 to 0.951, all above the 0.70 threshold (Hair et al., 2019), indicating excellent indicator reliability across AI Feedback Quality, AI Scaffolding Use, Creative Self-Efficacy, Instructional Design Ability, Professional Identity, and Self-Regulated Learning. Internal consistency was also robust, with Cronbach’s *α* values between 0.929 and 0.968 and CR values between 0.946 and 0.975, both exceeding recommended standards (Hair et al., 2019; Nunnally and Bernstein, 1994). Convergent validity was confirmed by AVE values of 0.778–0.888, all above 0.50 (Fornell and Larcker, 1981), as shown in Table 2.

**Table 2:** Convergent validity analysis table.

Construct	Item	Factor loading	Cronbach’s alpha	Composite reliability	Average variance extracted (AVE)
AI Feedback Quality	AIFQ1	0.926	0.962	0.970	0.867
	AIFQ2	0.942			
	AIFQ3	0.922			
	AIFQ4	0.940			
	AIFQ5	0.927			
AI Scaffolding Use	AISU1	0.936	0.968	0.975	0.888
	AISU2	0.942			
	AISU3	0.946			
	AISU4	0.936			
	AISU5	0.951			
Creative Self-Efficacy	CSE1	0.902	0.956	0.966	0.851
	CSE2	0.933			
	CSE3	0.941			
	CSE4	0.916			
	CSE5	0.921			
Instructional Design Ability	IDA1	0.919	0.960	0.969	0.862
	IDA2	0.933			
	IDA3	0.924			
	IDA4	0.947			
	IDA5	0.920			
Professional Identity in Early Childhood Education	PIEC1	0.882	0.929	0.946	0.778
	PIEC2	0.907			
	PIEC3	0.924			
	PIEC4	0.816			
	PIEC5	0.879			
Self-Regulated Learning	SRL1	0.917	0.961	0.969	0.864
	SRL2	0.931			
	SRL3	0.932			
	SRL4	0.938			
	SRL5	0.930			

Discriminant validity was assessed using both the Fornell–Larcker criterion and the heterotrait–monotrait ratio (HTMT). Table 3 demonstrates that the measurement model meets the Fornell–Larcker discriminant validity criterion. For all constructs, the square root of AVE (diagonal values)—AI Feedback Quality (0.931), AI Scaffolding Use (0.942), Creative Self-Efficacy (0.923), Instructional Design Ability (0.929), Professional Identity (0.882), and Self-Regulated Learning (0.930)—exceeds their correlations with other constructs. For example, Creative Self-Efficacy’s AVE square root (0.923) is higher than its correlations with all other variables (0.421–0.699), and Instructional Design Ability shows the same pattern (0.929 > 0.362–0.753).

**Table 3:** Discriminant validity analysis.

Construct	AI feedback quality	AI scaffolding use	Creative self-efficacy	Instructional design ability	Professional identity in early childhood education	Self-regulated learning
AI feedback quality	0.931					
AI scaffolding use	0.440	0.942				
Creative self-efficacy	0.528	0.421	0.923			
Instructional design ability	0.481	0.362	0.651	0.929		
Professional identity in early childhood education	0.448	0.405	0.529	0.591	0.882	
Self-regulated learning	0.575	0.481	0.699	0.753	0.653	0.930

Discriminant validity was also assessed using the Heterotrait–Monotrait ratio (HTMT). Following the recommendation of Henseler et al. (2015), HTMT values should be below 0.85 (or 0.90) to establish discriminant validity. The results indicated that all HTMT values were below the threshold, confirming adequate discriminant validity among the constructs, as shown in Table 4.

**Table 4:** HTMT results for discriminant validity.

Construct	AI feedback quality	AI scaffolding use	Creative self-efficacy	Instructional design ability	Professional identity in early childhood education	Self regulated learning
AI feedback quality						
AI scaffolding use	0.456					
Creative self-efficacy	0.550	0.436				
Instructional design ability	0.500	0.375	0.678			
Professional identity in early childhood education	0.472	0.422	0.558	0.620		
Self regulated learning	0.597	0.497	0.729	0.783	0.684	

Multicollinearity and common method bias were assessed using variance inflation factor (VIF) values. Following the criterion proposed by Kock (2015), VIF values below 3.3 indicate the absence of both multicollinearity and common method bias. The results showed that all VIF values ranged from 1.391 to 1.955, well below the recommended threshold. This suggests that multicollinearity is not a concern and that the model is free from common method bias (Table 5).

**Table 5:** Variance inflation factor (VIF) values.

Path relationship’	VIF
AI Feedback Quality → Creative Self-Efficacy	1.815
AI Feedback Quality → Self-Regulated Learning	1.815
AI Scaffolding Use → Creative Self-Efficacy	1.792
AI Scaffolding Use → Self-Regulated Learning	1.792
Creative Self-Efficacy → Instructional Design Ability	1.955
Professional Identity in Early Childhood Education → Creative Self-Efficacy	1.436
Professional Identity in Early Childhood Education → Self-Regulated Learning	1.436
Professional Identity in Early Childhood Education x AI Feedback Quality → Creative Self-Efficacy	1.391
Professional Identity in Early Childhood Education x AI Feedback Quality → Self-Regulated Learning	1.391
Professional Identity in_Early Childhood Education x AI Scaffolding Use → Creative Self-Efficacy	1.442
Professional Identity in Early Childhood Education x AI Scaffolding Use → Self-Regulated Learning	1.442
Self-Regulated Learning →Instructional Design Ability	1.955

### 4.3 Goodness of fit (GOF)

The Goodness of Fit (GOF) is an overall measure of model fit, with thresholds of 0.1 indicating weak fit, 0.25 indicating moderate fit, and 0.36 indicating strong fit (Vinzi et al., 2010). The GOF result for this study is 0.596, indicating a strong level of model fit.


$$\mathrm{GOF}=\sqrt{\left(\mathrm{AVE}\times R^{2}\right)}=\sqrt{\left(0.852\times 0.440\right)}=0.596$$

Model fit was also evaluated using PLS-SEM fit indices. The results indicated that the standardized root mean square residual (SRMR) values for the saturated and estimated models were 0.036 and 0.064, respectively, both below the recommended threshold of 0.08, suggesting a good model fit (Henseler et al., 2016), as shown in Table 6.

**Table 6:** SRMR model fit.

Fit index	Saturated model	Estimated model
SRMR	0.036	0.064

### 4.4 Path analysis

**Figure 2:**
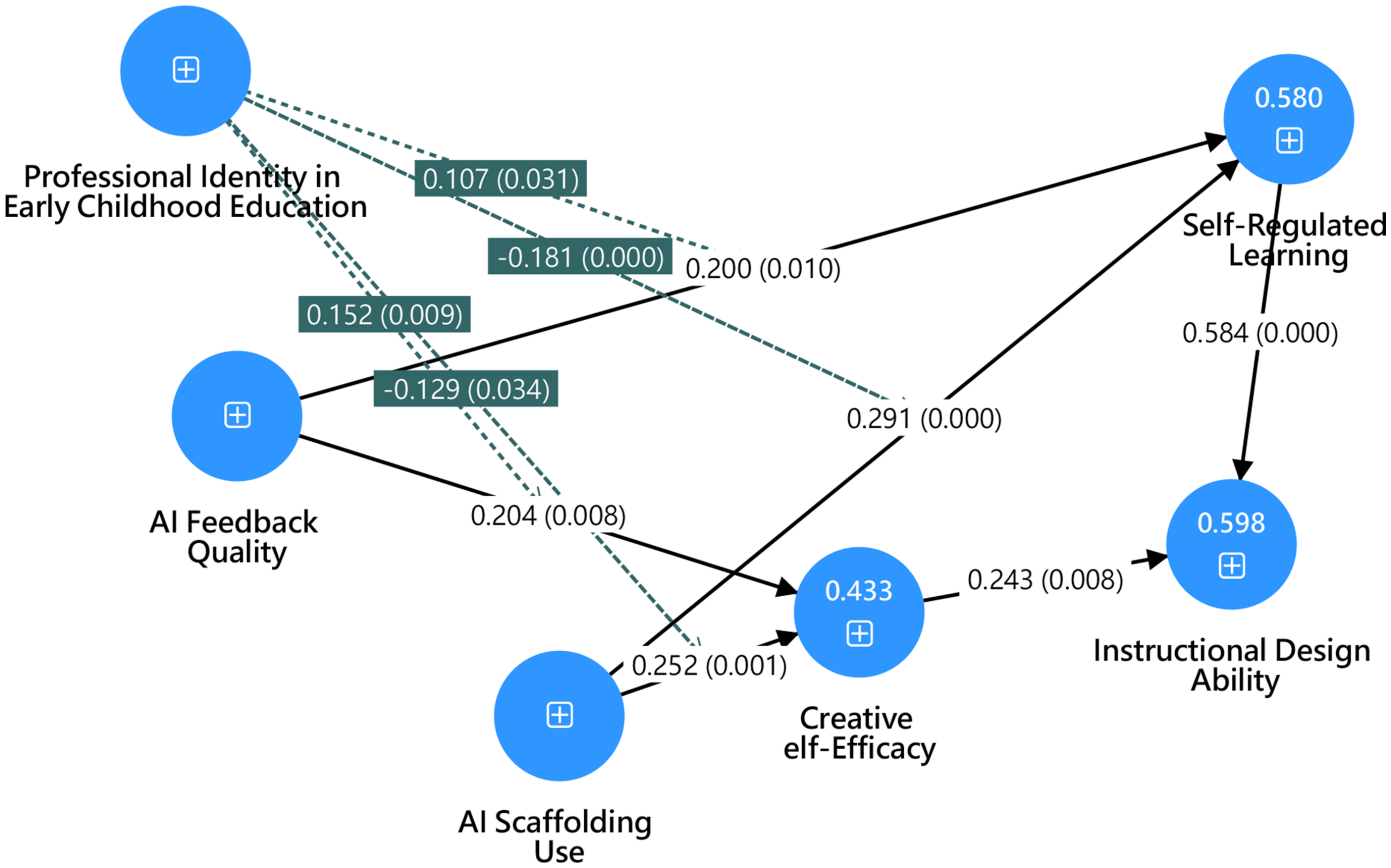
PLS-SEM statistical model diagram.

Table 7 and Figure 2 summarizes the structural model results, showing that all hypotheses (H1–H6) were supported. AI Feedback Quality positively influenced Self-Regulated Learning (*β* = 0.200, *p* = 0.010) and Creative Self-Efficacy (*β* = 0.204, *p* = 0.008), indicating that high-quality AI feedback enhances both learning regulation and creative confidence. AI Scaffolding Use also showed significant positive effects on Self-Regulated Learning (*β* = 0.291, *p* < 0.001) and Creative Self-Efficacy (*β* = 0.252, *p* = 0.001), highlighting the value of structured AI guidance. Self-Regulated Learning had the strongest impact on Instructional Design Ability (*β* = 0.584, *p* < 0.001), demonstrating that learners with stronger self-regulation skills perform better in instructional design tasks. Creative Self-Efficacy also positively predicted Instructional Design Ability (*β* = 0.243, *p* = 0.008).

**Table 7:** Path analysis.

No.	Path relationship	Original sample	Standard deviation	*t*-value	*p* values
H1	AI Feedback Quality → Self-Regulated Learning	0.200	0.077	2.583	0.010
H2	AI Feedback Quality → Creative Self-Efficacy	0.204	0.077	2.656	0.008
H3	AI Scaffolding Use → Self-Regulated Learning	0.291	0.054	5.354	0.000
H4	AI Scaffolding Use → Creative Self-Efficacy	0.252	0.078	3.234	0.001
H5	Self-Regulated Learning → Instructional Design Ability	0.584	0.087	6.691	0.000
H6	Creative Self-Efficacy → Instructional Design Ability	0.243	0.091	2.655	0.008

### 4.5 Mediation effects

Table 8 reports the mediation analysis results, showing that all indirect effects (H7–H10) were significant, as each 95% bootstrapped confidence interval excluded zero. For H7, AI Feedback Quality indirectly enhanced Instructional Design Ability through Self-Regulated Learning (*β* = 0.117, CI = 0.022–0.201). For H8, AI Scaffolding Use showed the strongest mediation effect through Self-Regulated Learning (*β* = 0.170, CI = 0.092–0.256). For H9, AI Feedback Quality also influenced Instructional Design Ability via Creative Self-Efficacy (*β* = 0.050, CI = 0.009–0.117). For H10, AI Scaffolding Use showed a smaller but still significant indirect effect through Creative Self-Efficacy (*β* = 0.061, CI = 0.020–0.126).

**Table 8:** Mediation effects analysis.

No.	Mediation effects	Original sample (O)	2.50%	97.50%
H7	AI Feedback Quality → Self-Regulated Learning → Instructional Design Ability	0.117	0.022	0.201
H8	AI Scaffolding Use → Self-Regulated Learning → Instructional Design Ability	0.170	0.092	0.256
H9	AI Feedback Quality → Creative Self-Efficacy → Instructional Design Ability	0.050	0.009	0.117
H10	AI Scaffolding Use → Creative Self-Efficacy → Instructional Design Ability	0.061	0.020	0.126

### 4.6 Moderation analysis

Table 9 shows that all four moderation hypotheses (H11–H14) were supported, indicating that Professional Identity significantly moderates the effects of AI Feedback Quality and AI Scaffolding Use. For H11, Professional Identity positively strengthened the effect of AI Feedback Quality on Self-Regulated Learning (*β* = 0.107, *p* = 0.031). For H12, however, Professional Identity weakened the effect of AI Scaffolding Use on Self-Regulated Learning (*β* = −0.181, *p* < 0.001). Similarly, for H13, Professional Identity amplified the influence of AI Feedback Quality on Creative Self-Efficacy (*β* = 0.152, *p* = 0.009). In contrast, H14 showed a negative moderation, with Professional Identity reducing the effect of AI Scaffolding Use on Creative Self-Efficacy (*β* = −0.129, *p* = 0.034).

**Table 9:** Moderation analysis.

No.	Moderation effects	Original sample (O)	Standard deviation (STDEV)	*T* statistics (|O/STDEV|)	*P*-values
H11	Professional Identity in Early Childhood Education x AI Feedback Quality → Self-Regulated Learning	0.107	0.050	2.153	0.031
H12	Professional Identity in Early Childhood Education x AI Scaffolding Use → Self-Regulated Learning	−0.181	0.042	4.287	0.000
H13	Professional Identity in Early Childhood Education x AI Feedback Quality → Creative Self-Efficacy	0.152	0.058	2.625	0.009
H14	Professional Identity in Early Childhood Education x AI Scaffolding Use → Creative Self-Efficacy	−0.129	0.061	2.116	0.034

## 5 Conclusions and discussion

### 5.1 Research conclusions

#### 5.1.1 Effects of AI feedback quality and AI scaffolding use on self-regulated learning and creative self-efficacy

This study confirms that both AI feedback quality and AI scaffolding use significantly enhance self-regulated learning and creative self-efficacy (H1–H4). These findings align with prior research in AI-supported education and further extend the theoretical understanding of these mechanisms.

First, high-quality AI feedback was found to significantly promote self-regulated learning (H1), echoing Afzaal et al. (2024) and Lan and Zhou (2025), who argue that specific and stimulating AI feedback enhances learners’ strategic awareness and metacognitive monitoring. Beyond existing evidence, this study further demonstrates that, within complex tasks such as instructional design, AI feedback can activate learners’ processes of identifying problems and adjusting strategies. This enriches the notion of “external regulatory resources” in SRL theory, positioning AI feedback as a critical *regulatory trigger t*hat initiates self-regulation.

Second, AI feedback quality was also found to positively influence creative self-efficacy (H2), consistent with Tierney and Farmer (2002) and McGuire et al. (2024). This suggests that high-quality feedback enhances learners’ confidence in tackling creative tasks through mechanisms such as mastery experiences, vicarious experiences, and social persuasion (Bandura, 1997). The present study further indicates that AI feedback not only serves an informational function but also provides psychological support. It can therefore be viewed as a situational efficacy enhancer, expanding the understanding of efficacy sources within Social Cognitive Theory.

Regarding scaffolding, the findings show that AI scaffolding use significantly improves self-regulated learning (H3), aligning with Hmelo-Silver et al. (2007) and Pea (2018), who emphasize that intelligent scaffolds help learners gradually internalize effective strategies. The task decomposition, examples, and step-by-step prompts provided by AI assist learners in integrating the phases of forethought, performance, and reflection, allowing the regulatory function of scaffolding theory to be more fully realized in AI-supported contexts.

Finally, AI scaffolding was also shown to enhance creative self-efficacy (H4), supporting Bandura’s (1997) and Jeong and Jeong’s (2025) assertions regarding the efficacy-building role of successful experiences and modeled examples. This study highlights that AI scaffolding reduces uncertainty in creative tasks and provides semantic cues and exemplars, enabling it to function both as a cognitive tool and a motivational tool for creativity. This dual role offers new extensions to both scaffolding theory and Social Cognitive Theory.

#### 5.1.2 Effects of self-regulated learning and creative self-efficacy on instructional design ability

The findings indicate that both self-regulated learning and creative self-efficacy significantly and positively predict preschool education majors’ instructional design ability (H5, H6). This not only aligns with existing research but also deepens the psychological process perspective within instructional design theory.

First, self-regulated learning exhibited a clear positive effect on instructional design ability, consistent with SRL models proposed by Zimmerman (2000a) and Pintrich (2004). Students with higher levels of SRL are better able to carry out forethought planning, strategic adjustment, and reflective revision throughout the instructional design process, enabling them to demonstrate more mature professional reasoning in complex tasks. This finding also aligns with AI-supported learning research (e.g., Banihashem et al., 2025), suggesting that students who possess strong self-monitoring and reflective skills are more capable of internalizing AI feedback and transforming it into higher-level design outputs. Thus, SRL is further validated as a foundational psychological mechanism supporting sophisticated professional tasks such as instructional design.

Second, the study confirms that creative self-efficacy positively predicts instructional design ability, supporting Tierney and Farmer’s (2002) and Jeong and Jeong’s (2025) proposed “creative efficacy–creative performance” pathway. Instructional design is a highly creative and open-ended task requiring integration of pedagogical principles, children’s characteristics, and innovative strategies. Students with higher CSE are therefore more likely to engage in exploration, apply diverse problem-solving approaches, and demonstrate greater persistence and creativity in design decisions. This finding also echoes McGuire et al. (2024), who argue that AI-supported environments enhance students’ performance in interdisciplinary and innovative tasks by boosting creative self-efficacy.

More importantly, the results reveal a complementary relationship between self-regulated learning and creative self-efficacy. Self-regulated learning provides the structural and strategic foundation for instructional design, whereas creative self-efficacy supplies the confidence and motivation needed to generate innovative solutions. This dual-process mechanism not only supplements Reigeluth’s (2013) emphasis on strategic processes within instructional design theory but also extends the TPACK framework (Koehler and Mishra, 2009) by highlighting that true professional design competence requires the integration of both strategic processes (SRL) and affective-creative processes (CSE).

Finally, this study offers new theoretical insights for AI-supported teacher education. The benefits of AI lie not only in improving the quality of students’ outputs but, more critically, in activating key psychological processes such as SRL and CSE, which in turn facilitate higher-level professional competence development. Building on these findings, this study proposes the AI-Triggered Instructional Design Model, which may serve as an important theoretical foundation for future research on AI integration in teacher education.

#### 5.1.3 Mediating effects of self-regulated learning and creative self-efficacy

This study confirms that self-regulated learning and creative self-efficacy both exert significant mediating effects between AI feedback quality, AI scaffolding, and instructional design ability (H7–H10). These findings indicate that the educational benefits of AI operate primarily through the activation of specific psychological processes rather than through direct effects alone.

First, the mediating effect of self-regulated learning (H7, H8) aligns with Zimmerman (2000a)and Pintrich (2004), and recent AI-supported learning research (Afzaal et al., 2024; Banihashem et al., 2025). These studies collectively argue that AI tools enhance learning outcomes by strengthening core SRL capacities such as strategic awareness, monitoring, and reflection. This study further demonstrates that, within highly complex instructional design tasks, both AI feedback and AI scaffolding help students better analyze task requirements, adjust strategies, and improve the quality of their design products by facilitating SRL processes. Notably, the indirect effect of AI scaffolding → SRL → instructional design ability (H8) was the strongest, supporting the arguments of Hmelo-Silver et al. (2007) and Pea (2018) that intelligent scaffolds reinforce strategic learning. This study provides the first empirical evidence of this effect in the context of early childhood teacher education.

Second, creative self-efficacy also exhibited significant mediating effects (H9, H10), consistent with the “creative efficacy–creative performance” mechanism proposed by Tierney and Farmer (2002) and Jeong and Jeong (2025). The findings confirm that AI feedback and scaffolding enhance students’ creative confidence, encouraging them to explore new ideas, make iterative refinements, and adopt innovative strategies. This result also extends Bandura’s (1997) model of efficacy sources by showing that the examples, semantic cues, and positive feedback provided by AI can function as AI-mediated efficacy signals, offering a new perspective on how efficacy beliefs are formed.

Finally, the study reveals that the mediating effect of self-regulated learning is stronger than that of creative self-efficacy, highlighting the strategic and reflective nature of instructional design as a professional task. This finding aligns with Reigeluth’s (2013) assertion that strategic competence plays a more central role in instructional design performance. Nonetheless, creative self-efficacy, though relatively weaker, remains an important motivational driver of students’ engagement and creative outcomes. Based on these insights, this study proposes a dual-process mediation model for AI-supported instructional design, illustrating how AI tools simultaneously influence students’ professional competence through strategic (SRL) and affective–creative (CSE) pathways.

#### 5.1.4 Moderating effects of professional identity in early childhood education

This study reveals a theoretically meaningful and clearly patterned dual moderating effect of professional identity in AI-supported learning. First, professional identity strengthens the positive effects of AI feedback quality on self-regulated learning and creative self-efficacy (H11, H13). When students strongly identify with their professional role in early childhood education, they are more likely to perceive AI-generated analyses and recommendations as authoritative and developmentally valuable. This heightened credibility increases their likelihood of engaging in strategic reflection, adjusting their abilities, and enhancing their creative confidence. These findings align with identity theory and Bandura’s conceptualization of efficacy sources, suggesting that professional identity facilitates the absorption and internalization of external positive cues.

In contrast, professional identity was found to weaken the positive effects of AI scaffolding on self-regulated learning and creative self-efficacy (H12, H14). This negative moderation indicates that students with stronger professional identity tend to rely less on procedural, step-by-step guidance provided by AI, instead preferring their own professional intuition and creative judgment. This pattern is particularly salient within the context of Mainland China’s teacher education system, where professional identity is often shaped through structured training and normative expectations, reinforcing learners’ preference for internally guided pedagogical judgment. This suggests that the standardized structure of AI scaffolds may conflict with the autonomy and creative space valued by learners with higher professional competence, reducing the scaffolding’s effectiveness. This pattern aligns with prior research on teacher technology adoption, which shows that individuals with higher levels of professionalism prefer self-directed adjustment rather than dependence on external scaffolds.

Overall, the results indicate that the effectiveness of AI tools does not stem solely from the tools themselves, but rather from their professional congruence. AI feedback that is professionally rich and aligned with role expectations enhances the motivation of students with strong professional identity, whereas highly procedural AI scaffolds may create tension with their sense of autonomy and professional confidence. Based on these findings, this study proposes a Professional Congruence Moderation Model, offering a new theoretical lens for the field of AI and educational psychology and highlighting the need for educators to tailor the type and level of AI support according to students’ levels of professional identity.

### 5.2 Discussion

#### 5.2.1 Academic contributions

The findings should be interpreted as reflecting theoretically grounded associations rather than definitive causal relationships, within the specific context of Mainland China’s teacher education system, which is characterized by structured training, standardized curricula, and norm-oriented professional development. First, this study develops a dual psychological process model of AI-supported learning, addressing the limitations of prior research that has largely emphasized technical effectiveness. The findings demonstrate that AI feedback and AI scaffolding influence instructional design ability not merely through surface-level learning outcomes but indirectly through two key psychological processes—self-regulated learning (SRL) and creative self-efficacy (CSE). This expands the common tool-centered perspective in AI education research. The proposed model positions AI as a *psychological facilitator* that activates strategic awareness and creative belief, thereby clarifying the deeper psychological mechanisms underpinning AI-supported learning.

Second, this study extends self-regulated learning theory and Social Cognitive Theory to the context of AI-supported teacher education. The results show that AI feedback and scaffolding function as external regulatory stimuli that promote learners’ monitoring, reflection, and strategy adjustment, echoing SRL theory’s emphasis on external supports. Moreover, the study provides evidence that AI can offer efficacy-building signals—such as modeled success and linguistic persuasion—thereby enhancing creative self-efficacy. This extends Bandura’s (1997) model of efficacy sources into what may be considered an AI-mediated efficacy source, offering new theoretical applications of classic psychological frameworks in teacher education and AI-integrated learning environments.

Third, this study identifies a dual moderating mechanism of professional identity in determining the effectiveness of AI support. The results show that students with strong professional identity respond more positively to professionally rich AI feedback, yet may benefit less from highly procedural AI scaffolding due to their preference for autonomy and professional judgment. This challenges the linear assumption that stronger professional identity always enhances the effectiveness of AI tools. Instead, the findings highlight that AI effectiveness depends on its alignment with the learner’s professional identity. The proposed Professional Congruence Moderation Model provides a new theoretical foundation for understanding the interaction between AI tools and professional identity.

The negative moderating effect of professional identity on the relationship between AI scaffolding and learning outcomes cannot be sufficiently explained by a general preference for autonomy alone. Rather, this phenomenon should be understood in light of the distinctive professional characteristics of early childhood education (ECE) and the nature of pedagogical expertise development. First, early childhood education emphasizes context-sensitive, child-centered, and flexible instructional practices. Unlike highly structured disciplines, ECE requires preservice teachers to continuously adapt instructional strategies based on children’s developmental needs, emotional responses, and situational dynamics. As a result, learners with strong professional identity tend to develop a pedagogical belief system that prioritizes responsiveness, creativity, and professional judgment over procedural guidance (Beauchamp and Thomas, 2009; Tondeur et al., 2017). In this context, highly structured AI scaffolding may be perceived as overly prescriptive, thereby constraining pedagogical flexibility rather than supporting it.

Second, from the perspective of Self-Determination Theory (Deci and Ryan, 2000), professional identity strengthens internalized motivation and autonomy at a deeper level. However, autonomy in this context is not merely a preference for independence, but reflects a higher-order need for self-authorship and professional agency. When AI scaffolding provides step-by-step procedural guidance, it may inadvertently undermine this sense of agency, particularly for learners who already possess a well-developed professional self-concept. This aligns with prior research suggesting that experienced or identity-strong learners are more likely to resist externally imposed structures that conflict with their internalized standards of practice.

Third, instructional design in early childhood education is inherently an ill-structured and creative task, requiring the integration of pedagogical knowledge, emotional understanding, and situational judgment. In such tasks, excessive scaffolding may reduce cognitive flexibility and limit divergent thinking, thereby weakening its effectiveness for learners with high professional identity. In contrast, these learners may benefit more from less structured, feedback-oriented AI support that allows them to exercise professional reasoning. Therefore, the negative moderating effect observed in this study reflects a misalignment between the procedural nature of AI scaffolding and the autonomy-oriented, context-sensitive professional cognition of ECE students. This finding highlights that the effectiveness of AI support is contingent not only on its availability but also on its congruence with learners’ professional identity and epistemological beliefs.

#### 5.2.2 Practical contributions

First, this study provides concrete evidence for integrating AI feedback and AI scaffolding into teacher preparation programs. The findings demonstrate that both forms of AI support effectively enhance early childhood education students’ self-regulated learning and creative self-efficacy, thereby improving their instructional design ability. This suggests that AI can serve as a valuable learning support tool within teacher education. AI feedback, in particular, shows strong effectiveness in promoting reflection, revision, and creative expression, making it a useful supplement for lesson plan development, microteaching activities, and assignment feedback—especially in contexts where human feedback requires substantial time and workload.

Second, the results help guide educational practice toward more precise use of AI to strengthen instructional design ability. As AI exerts its effects primarily through SRL and CSE, educators should design AI-supported activities that simultaneously cultivate strategic awareness (e.g., monitoring and evaluation) and creative confidence. For example, instructors may incorporate tasks such as AI-assisted feedback analysis or AI-supported collaborative design within lesson-planning courses, enabling students to practice reflection and creative generation under AI guidance and thereby maximize AI’s impact on instructional design competence.

Third, the study offers practical guidelines for course designers in choosing and applying AI tools. Because professional identity moderates the effects of AI feedback and AI scaffolding differently, AI implementation should consider students’ level of professional development. Students with stronger professional identity benefit more from analytically rich AI feedback but may rely less on procedural AI scaffolds. This implies that AI tool selection should be aligned with course goals: AI feedback is preferable in courses emphasizing professional judgment and creative quality, whereas AI scaffolding remains beneficial in foundational training or demonstration-based learning. These insights help educators allocate AI tools more strategically to enhance instructional effectiveness.

Fourth, the findings support the development of teacher education models in which AI and professional identity construction reinforce each other. Since AI’s effectiveness varies according to students’ level of professional identity, teacher education programs should integrate AI use with professional identity-building activities. For example, courses that stress early childhood education values may incorporate discussions based on AI-generated feedback, allowing AI to become part of students’ professional reasoning. In skill-oriented courses, AI scaffolding can be used to support procedural practice. Such an approach prevents overreliance on or resistance to AI and promotes a more balanced and sustainable model of AI-supported teacher preparation.

### 5.3 Limitations and future research

This study has several limitations. First, the use of a cross-sectional survey design limits the ability to draw definitive causal inferences among the variables. Although the proposed relationships are grounded in established theoretical frameworks, the findings should be interpreted as theoretically supported associations rather than strictly causal relationships. In addition, AI-supported learning is inherently a dynamic and iterative process involving continuous feedback, reflection, and adaptation, which cannot be fully captured through a one-time measurement. Future studies may adopt longitudinal or experimental designs to better examine causal mechanisms and the temporal evolution of AI-supported learning processes. Second, the sample was limited to early childhood education undergraduates in Mainland China, which reflects a specific teacher education context characterized by structured training systems and standardized professional development pathways. While this context provides a meaningful setting for examining AI-supported learning, it may limit the generalizability of the findings to other educational systems (e.g., Taiwan or Western contexts), where teacher education structures and professional identity formation processes differ. While this distribution reflects the structural characteristics of early childhood education, it may limit the generalizability of the findings across more gender-diverse educational technology contexts. Third, AI feedback and scaffolding were assessed through self-reported measures, without incorporating actual interaction data or distinguishing between different types of AI tools. Future studies may integrate behavioral logs or analyses of generative AI interactions to enrich the understanding of AI-supported learning. Moreover, this study focused on self-regulated learning and creative self-efficacy, leaving out other relevant psychological processes such as cognitive load or trust in AI. Finally, the positive and negative moderating effects of professional identity suggest a complex underlying mechanism that warrants further empirical investigation. Overall, future research can deepen the proposed model through methodological diversification, broader sampling, and expanded variable exploration.

## Data Availability

The datasets generated by the survey research during and/or analyzed during the current study are available in the Zenodo data repository, https://doi.org/10.5281/zenodo.18062254.
